# Silicon foil patching for blast tympanic membrane perforation: a retrospective study

**DOI:** 10.3325/cmj.2019.60.503

**Published:** 2019-12

**Authors:** Srećko Branica, Krsto Dawidowsky, Lana Kovač-Bilić, Mario Bilić

**Affiliations:** Department of Otolaryngology and Head and Neck Surgery, University Hospital Center Zagreb, School of Medicine, University of Zagreb, Zagreb, Croatia

## Abstract

**Aim:**

To establish whether covering the tympanic membrane perforation after war blast injury with silicon foil can enhance the ear drum healing rate and to determine the appropriate timing of silicon patching.

**Methods:**

We retrospectively analyzed the charts of 210 patients wounded during the Homeland War in Croatia 1991-1995, with 315 blast tympanic membrane perforations. In 44 patients (61 perforations), the eardrum perforation was covered by silicon foil, whereas in 166 patients (254 perforations) it was left to heal spontaneously. The patients who underwent the patching procedure were divided in two groups according to the time period between the blast injury and the procedure: 38 perforations were treated within 3 days and 23 perforations were treated 4 to 6 days after the blast injury.

**Results:**

The rate of tympanic membrane healing in the silicon foil patching group was significantly higher (91.8%) than that in the group of perforations left to heal spontaneously (79.9%, *P* = 0.029). The healing rate was significantly higher in the group treated within 3 days after the blast injury (97.4%) than in the group treated 4 to 6 days after the injury (82.6%, *P* = 0.042).

**Conclusion:**

Covering the perforation after the war blast injury with silicon foil significantly improves the rate of tympanic membrane healing. To obtain the best healing outcome, the procedure should be performed within the first 72 hours after the trauma.

Correspondence to: Srećko Branica Department of Otolaryngology and Head and Neck Surgery University Hospital Center Zagreb Kišpatićeva 12 KBC Zagreb  *sbranica@mef.hr*

## 

Explosions produce an instantaneous positive wave of air pressure, which can disrupt or perforate the ear drum, and in the case of an extreme blast, dislocate or interrupt the ossicular chain, rupture the round window, or displace the stapes footplate. A pressure of 35 kPa can damage the tympanic membrane, while a blast stronger than 100 kPa can rupture more than 50% of the healthy ear drum. The extent of the injury can also be influenced by the strength of the explosion and distance from it. Tympanic membrane trauma is more severe if the explosion occurs indoors, since the positive air wave can be reflected from the surrounding walls (1-5). After the blast injury, there is also an increased risk of middle ear infection and cholesteatoma development, since remnants of the tympanic membrane can easily grow into the middle ear mucosa (6).

The majority of tympanic membrane perforations heal spontaneously; however, the healing rate greatly varies in various studies (38%-94%) (7-10). To increase the rate of tympanic membrane healing some authors recommend early myringoplasty, while others leave the perforation to heal spontaneously and perform surgery only in a case of failure (11,12). The success rate of the surgical procedure is slightly higher in the cases of traumatic ear drum perforation than in the cases of chronic otitis media. This can be explained by the fact that in traumatic perforations, the normal function of the Eustachian tube is preserved and there is a lower infection rate (13).

In order to enhance spontaneous healing after the traumatic perforation of the tympanic membrane, different non-surgical methods of patch-covering and various materials were proposed: paper, gelatin sponge, micropore strip tape, silk, and eggshell membrane. Although studies applying these methods reported improved healing, all of them involved a limited number of cases (10,14-16).

Middle ear surgery regularly employs silicon foil for tympanic membrane and meatal skin covering after tympanoplasty (17). In our study, we assessed the use of silicon foil as a patching material for the ruptured ear drum to determine the healing rate of the tympanic membrane after the blast injury. The study had two aims: 1) to explore the effect of silicon foil application on TM healing process after blast injury, and 2) to determine the best timing for silicon foil patching to achieve the best healing results.

## PARTICIPANTS AND METHODS

### Patients

During the War in Croatia 1990-1995, a total of 1054 blast injuries of the ear, 549 of which were tympanic membrane perforations, were treated in the Department of Otolaryngology and Head and Neck Surgery, University Hospital Center Zagreb. In 63 cases, when less than 50% of the eardrum was involved within the perforation and when patients accepted the procedure, we performed repositioning of the inverted perforation edges and covered the perforation with silicon foil. In other cases, we left the perforations either to heal spontaneously (the smaller ones, 358 cases) or performed tympanoplasty (in cases of larger perforations, if more than 50% of the ear drum was perforated). Out of 63 perforations in which edges repositioning and silicon foil application were performed, 2 were lost to the 6-month follow-up. At the end of the study, the treatment group consisted of 44 patients with 61 perforations. From the group of 358 untreated perforations, 104 patients were excluded because they came to the hospital more than 6 days after the blast injury or were lost to follow-up. The control group comprised the remaining 254 perforations (in 166 patients).

The inclusion criteria were the performance of the procedure within 6 days after the blast injury, regular postoperative clinical and audiological follow-up, and follow-up period longer than 6 months. Exclusion criteria were injuries with dislocation or interruption of the ossicular chain and previous ear disease.

The treatment group comprised 43 men and one woman, aged 19-52 years (mean 28.7 ± 7.3 years). The right ear was injured in 31 (50.8%) and the left ear in 30 patients (49.2%). The control group comprised 163 men and 3 women in whom perforations were left to heal spontaneously, aged 19-58 years (mean 28.2 ± 8.9 years). The right ear was injured in 126 (49.6%) and the left ear in 128 patients (50.4%).

During the first examination, before the edge repositioning, we determined the perforation size. Depending on the perforation size, the perforations were divided into three groups: up to 10% of the tympanic membrane surface, from 11% to 25% of the tympanic membrane surface, and from 26% to 50% of the tympanic membrane surface. Considering the average size of the human eardrum (measuring 9-10 mm vertically and 8-9 mm horizontally), perforations covering up to 10% of the tympanic membrane surface were smaller than 2.5 mm in diameter, perforations covering from 11% to 25% measured from 2.6 mm to 4 mm, while perforations covering from 26% to 50% measured 4.1 mm to 5.9 mm. Perforations larger than 50% of the tympanic membrane surface were not treated by the repositioning of inverted edges and silicon foil application.

To determine the optimal moment for silicone foil placement, we also divided our patients in two groups considering the time elapsed from the blast injury. In the first group of 38 perforations, edge repositioning and foil covering were performed within 3 days from the blast injury, while in the group of 23 perforations the procedures were performed 4 to 6 days after the blast injury. The study was approved by the Ethics Committee of the University Hospital Centre Zagreb (02/21 AG). All patients provided informed consent.

### Methods

To facilitate the spontaneous perforation healing, silicon foil (0.13 mm thickness) was positioned over the tympanic membrane, covering the perforation and a few millimeters of the ear drum around the perforation edge. All cases with inverted perforation edges underwent microscopical repositioning of the epithelial layer of the eardrum in local anesthesia by transmeatal approach. At the end of the procedure, the external auditory meatus was packed with resorptive gelfoam sponge for silicon foil fixation. The procedure was done within the first few days after the blast injury. All patients received 1500 mg of amoxicillin (Amoxil, Pliva, Zagreb, Croatia) divided in three daily doses as antibiotic prophylaxis for the first 5 days after the procedure. In perforations covering up to 10% of the tympanic membrane surface, the silicon foil was left for 2 months, in those covering 11%-25% of the tympanic membrane surface it was left for 3 months, and in those covering 26%-50% of the tympanic membrane surface it was left for 4 months. After silicon foil removal, the healing process of the ear drum was controlled monthly with the microscope. To additionally confirm the perforation closure, 6 months after the procedure all patients underwent tympanometry. Before the procedure and 6 months after it, we performed pure tone audiometry (PTA) to determine the patients’ hearing level. If the perforation persisted for longer than 6 months, we considered it unhealed. In all cases of incomplete healing 6 months after the blast injury, we performed tympanoplasty.

All controls were also treated conservatively with 1500 mg of amoxicillin (Amoxil, Pliva) divided in three daily doses as antibiotic prophylaxis for the first 5 days.

### Statistical analysis

Descriptive statistics was used to summarize the data. To determine if there were significant differences between treatment and control group and between subgroups we used the χ^2^ test. To evaluate the relationships between the perforation size and the air-bone gap we used Spearman correlation. Statistical analysis was performed in SPSS, version 12.0 (SPSS Inc., Chicago, IL, USA). The level of significance was set at *P* lower than 0.05.

## RESULTS

### The success of the technique

A total of 56/61 (91.8%) perforations in the treatment group and 203/254 perforations in the control group (79.9%) healed completely, with a significant difference between the groups (chi square test, χ^2^ = 4.751, *P* = 0.029). During the 6-month follow-up period, 3 cases in the treatment group (4.9%) and 41 cases in the control group (16.1%) had purulent suppuration from the ear, with a significant difference between the groups (chi square test, χ^2^ = 5.156, *P* = 0.023).

The average conductive hearing loss (air-bone gap) in the treatment group was 17.0 ± 10.2 dB, measured by PTA before the procedure. Six months after the treatment, air-bone gap was 5.4 ± 5.6 dB, with an absolute recovery rate of 11.6 ± 8.9 dB. The initial conductive hearing loss significantly correlated with the perforation size in the group treated immediately after the blast injury (Spearman's correlation coefficient R = 0.480, *P* < 0.001). The correlation between the perforation size and final air-bone gap in dB was also significant (Spearman's correlation coefficient R = 0.342, *P* = 0.007), as well as the correlation between the perforation size and air-bone gap recovery (Spearman's correlation coefficient R = 0.276, *P* = 0.031).

### Influence of patching time on healing rate

In 38 perforations (62.3%), the foil covering was performed within 72 hours from the blast injury, and in the remaining 23 perforations (37.7%) 4 to 6 days after the injury. Among the perforations treated within the first three days, 37 out of 38 healed completely (97.4%). Among perforations treated 4 to 6 days after the injury, 19 out of 23 (82.6%) healed completely (Table 1).

**Table 1:** The total number of perforations and total number of healed perforations (controlled by otomicroscopy and confirmed by tympanometry) according to the perforation size and time from the blast injury to silicon foil patching

Percentage of the perforated tympanic membrane surface	Perforations treated within 3 days after the blast injury		Perforations treated from day 4 to day 6 after the blast injury		Total number of perforations	Total number of healed perforations
	number of perforations	number of healed perforations	number of perforations	number of healed perforations		
≤10%	8	8	6	5	14	13
11-25%	16	16	10	9	26	25
26-50%	14	13	7	5	21	18
Total	38	37/38	23	19/23	61	56/61

The proportion of healed perforations treated within the first three days was significantly higher than the proportion of healed perforations treated 4 to 6 days after the injury (chi square test, χ^2^ = 4.148, *P* = 0.042). The proportion of healed perforations was also significantly higher in the group treated within the first 3 days than in the control group (chi square test, χ^2^ = 6.874, *P* = 0.009). Although the proportion of healed perforations in the group treated 4-6 days after the blast injury was higher than in the control group, the difference was not significant (chi square test, χ^2^ = 0.096, *P* = 0.757).

## DISCUSSION

This study showed that silicon foil application with perforation edges repositioning significantly improved the healing of a ruptured tympanic membrane. To the best of our knowledge, this is the first report on the effects of eardrum perforation healing when silicone sheets are used after war blast injuries of the tympanic membrane.

Refreshing and everting of perforation edges, if systematically performed together with patch-covering of ear drum defects, facilitates the healing of a ruptured tympanic membrane (9,10). Our study confirmed this healing effect on a sample of blast injuries of the ear drum. Additionally, we showed that silicon foil tympanic membrane patching and gel-foam packing of the external meatus decreased the possibility of ear infection in blast injury cases. This procedure resulted in a significantly lower proportion of suppuration in the treatment group compared with the spontaneous healing group.

The perforated tympanic membrane heals and inverted perforation edges grow into the middle ear mucosa within a few days. Therefore, the successful repositioning of the perforation edges is harder to perform as the time from the injury increases and the risk of complete eardrum healing decreases.

In short, tympanic membrane perforations after the blast injury heal significantly better if the treatment is applied; moreover, the healing rate increases if the procedure is performed earlier. We believe that the procedure leads to better healing because it prevents the inverted epithelial edges concrescence to the inner mucous layer of the tympanic membrane, facilitates membrane growth with silicone foil, and decreases the infection rate after the procedure.

The average conductive hearing loss in the treatment group early after the blast injury was 17.0 dB, while after the treatment it was 5.4 dB, with a recovery rate of 11.6 dB. These results show a slightly better hearing improvement compared with some other studies (7-9). This difference can be explained by the exclusion of the largest perforations, which led to a profound hearing loss, from our research. Our study showed a significant correlation between the perforation size and the air-bone gap values in dB. The highest correlation was observed between the perforation size and the initial conductive hearing loss, and the lowest between the perforation size and the air-bone gap recovery. Contrary to our expectations, the hearing level improved in correlation with the perforation size only in small and medium sized perforations, but not in the largest perforations with severe conductive hearing loss. The reason for this might be the highest rate of unhealed perforations in this group of patients, which influenced the statistical results.

Although limited by retrospective design and a small number of patients, the study showed that the application of silicon foil with repositioning of the inverted edges of the tympanic membrane rupture significantly decreased the incidence of an infection and increased the rate of ear drum healing, reducing the need for tympanoplasty. It is a simple procedure that can be performed in a local anesthesia, and we recommend it not just for the cases of a blast injury, but also for all other cases of traumatic ear drum perforation. To achieve the best healing results, the patients should be treated early after the injury, if possible within the first 72 hours.
